# Improved high-dimensional prediction with Random Forests by the use of co-data

**DOI:** 10.1186/s12859-017-1993-1

**Published:** 2017-12-28

**Authors:** Dennis E. te Beest, Steven W. Mes, Saskia M. Wilting, Ruud H. Brakenhoff, Mark A. van de Wiel

**Affiliations:** 1 Department of Epidemiology and Biostatistics, VU University Medical Center, Amsterdam, 1007 MB The Netherlands; 2 Department of Mathematics, VU University, Amsterdam, 1081 HV The Netherlands; 3 Department of Otolaryngology-Head and Neck Surgery, VU University Medical Center, Amsterdam, 1007 MB The Netherlands; 4 Department of Medical Oncology, Erasmus MC Cancer Institute, Erasmus University Medical Center, Rotterdam, 3015 CE The Netherlands

**Keywords:** Classification, Random forest, Gene expression, Methylation, DNA copy number, Prior information

## Abstract

**Background:**

Prediction in high dimensional settings is difficult due to the large number of variables relative to the sample size. We demonstrate how auxiliary ‘co-data’ can be used to improve the performance of a Random Forest in such a setting.

**Results:**

Co-data are incorporated in the Random Forest by replacing the uniform sampling probabilities that are used to draw candidate variables by co-data moderated sampling probabilities. Co-data here are defined as any type information that is available on the variables of the primary data, but does not use its response labels. These moderated sampling probabilities are, inspired by empirical Bayes, learned from the data at hand. We demonstrate the co-data moderated Random Forest (CoRF) with two examples. In the first example we aim to predict the presence of a lymph node metastasis with gene expression data. We demonstrate how a set of external *p*-values, a gene signature, and the correlation between gene expression and DNA copy number can improve the predictive performance. In the second example we demonstrate how the prediction of cervical (pre-)cancer with methylation data can be improved by including the location of the probe relative to the known CpG islands, the number of CpG sites targeted by a probe, and a set of *p*-values from a related study.

**Conclusion:**

The proposed method is able to utilize auxiliary co-data to improve the performance of a Random Forest.

**Electronic supplementary material:**

The online version of this article (doi:10.1186/s12859-017-1993-1) contains supplementary material, which is available to authorized users.

## Background

High-dimensional prediction is inherently a difficult problem. In this paper we demonstrate how to improve the performance of the Random Forest (RF) on high-dimensional (in particular genomics) data by guiding it with ‘co-data’. Here, co-data is defined as any type of qualitative or quantitative information on the variables that does not use the response labels of the primary data. The primary data may, for example, be a set of gene expression profiles with corresponding binary response labels. Examples of co-data are: *p*-values on the same genes in a external, related study, correlations with methylation or DNA copy number data, or simply the location on the genome. Guiding a prediction model by co-data may lead to improved predictive performance and variable selection.

Several methods are able to incorporate co-data during model training. A general multi-penalty approach was suggested by [1], a weighted lasso by [2], and a group-regularized ridge by [3]. These methods are all based on penalized regression, with a penalty parameter that is allowed to vary depending on the co-data, effectively rendering co-data based weights. The group-lasso [4] and sparse group-lasso [5] are also regression-based, but these methods apply a specific group-penalty that can exclude entire groups of variables. Except for the group-regularized ridge, all these methods allow for only one type of co-data. In addition, except for the weighted lasso, these methods require the co-data to be specified as groups. The weighted lasso can handle one source of continuous co-data, but requires an assumption about the functional form of the penalty weighting and the co-data. For some types of co-data this functional form is largely unknown. Hence, it may be desirable to learn it from the co-data, and to enforce monotonous weights to ensure stability and interpretability.

The Random Forest (RF) is a learner that is popular due to its robustness to various types of data inputs, its ability to seamlessly handle non-linearities, its invariance to data transformations, and its ease of use without any or much tuning [6]. The RF is suitable and computationally efficient for genomics data, with typically the number of variables, *P*, largely exceeding the sample size, *n* [7, 8]. Its scale invariance makes it a good candidate to analyse RNASeq data. Due to the skewed nature of such data, their analysis is less straightforward with penalized regression techniques and results depend strongly on the data transformation applied [9]. Our aim is to develop a co-data moderated RF (CoRF) which allows the joint use of multiple types of co-data, the use of continuous co-data, and flexible modeling of the co-data weights. Conveniently, these co-data are only used when training the classifier; they are not required for applying the classifier to new samples.

The described methodology can in principle be used with any bagging classifier that uses the random subspace method [10], but in this paper we focus on the RF. The method is exemplified with two examples. First, we aim to predict the presence of a lymph node metastasis (LNM) for patients with head and neck squamous cell carcinoma (HNSCC) using TCGA RNAseq data. We show how the use of several types of co-data, including DNA copy number, an external gene signature and mRNA microarray data from an independent patient cohort, improves the predictive performance, and validate these results on a second independent data set. The computational efficiency of the method is illustrated with a second example, where our aim is to predict the last precursor stage for cervical cancer based on methylation data with very large *P*≈350.000. The co-data in this example consists of the location of the methylation site, the number of CpGs, and the external set of *p*-values.

## Methods and Results

### Random forest

The aim of a supervised RF is to predict per sample *i,i*=1,…,*n*, an outcome *Y*_*i*_ using a set of variables *X*_*ij*_ where *j*=1,…,*P* indicates the variables. Here, we focus on binary outcome *Y*_*i*_, although the entire methodology and software also applies to continuous and censored (e.g. survival) outcomes. A RF consists of a large number of unpruned decision trees, where each tree is grown on a bootstrap sample of the data. At each node split in each tree only a random subset of the variables are candidates, its size denoted by *mtry*, typically set at $\sqrt P$. In a standard RF, all variables have an equal probability of being candidates. Predictions are issued by majority voting across all trees, or on a fractional scale (fraction of trees predicting *Y*_*i*_=1). We will use the latter for assessing predictive performance. A RF is fitted to a bootstrap sample of the data implying that per tree the remaining fraction (on average 0.368) is out-of-bag (oob) and can be used to obtain an estimate of the prediction error. This leads to a computational advantage compared to methods that require cross-validation for this purpose.

### Group-specific probabilities

We first briefly describe our method using one source of grouped co-data only. Here, the basic idea is that, when an a priori grouping of variables is available (co-data), we may sample the variables according to group-specific probabilities, and these probabilities can be estimated empirically from the data. When the number of groups is limited, only a few parameters need to be estimated (the group specific probabilities). Especially when the difference in predictive power between groups of variables is large, the predictive performance may be enhanced.

In practice, this means we first need to run a base RF (i.e. uniform sampling probabilities). From this initial fit, we obtain the number of times each variable is used across all trees. Then, the new group-specific probabilities *w*_*g*_ are:

1 $$ w_{g} = \left(\hat{p}_{g}^{\normalsize{sel}} - \gamma p^{0}\right)^{+},  $$

where $\hat {p}_{g}^{\normalsize {sel}}$ is the proportion of selected variables from group *g* across all trees divided by the size of group *g* and *p* ^0^=1/*P* is the expected value of $\hat {p}_{g}^{\normalsize {sel}}$ when the group structure is uninformative. Parameter *γ* can be used to tune the RF to adapt to group-sparsity by thresholding, but may also be set to one to avoid tuning. After normalizing *w*_*g*_ such that these sum to one across variables, we obtain sampling probabilities $\tilde {w_{g}}$. Then, a new RF is trained using these probabilities instead of the uniform ones, rendering the CoRF.

### Model-based probabilities

**Fig. 1:**
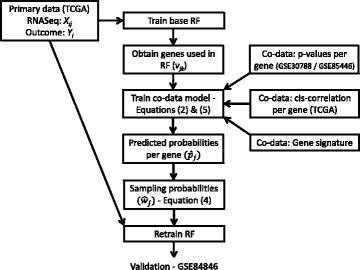
Illustration of the sources of data used in CoRF for the LNM example. First, a base RF is fitted on the training data. Its output, *v*_*ij*_, together with the co-data, is used to train the co-data model. From the co-data model, we obtain a probability per gene used for refitting on the training data. In an extra step we validate the results on GSE84846

Next we extend the described method to allow for multiple sources of co-data, including continuous co-data. Figure 1 schematically displays the method for the first application. First, we enumerate all node splits in all trees. Then, we define *v*_*jk*_ as a binary variable indicating whether or not variable *j* was used in the *k* ^*th*^ split, and $V_{j} = \sum _{k} v_{jk}$ as the total number of times that variable *j* was used.

The main challenge in modeling multiple types of co-data, is that the various types of co-data may be collinear. We therefore need to de-tangle how well the various types of co-data explain *v*_*jk*_. For that, we use a co-data model. We propose to use the logistic regression framework for this. We denote the *P*×*C* co-data design matrix by *X*, where *X*_*jc*_ contains the co-data information for the *j*th variable and the *c*th co-data type, and where nominal co-data on *L* levels is represented by *L*−1 binary co-data variables. Then, *v*_*jk*_ is Bernoulli distributed with *v*_*jk*_∼*Bern*(*p*_*j*_), and we estimate *p*_*j*_ using a logistic regression:

2 $$ \text{logit}(p_{j}) = \alpha_{0} + \sum_{c=1}^{C} X_{jc}\alpha_{c}  $$

From the co-data model, we obtain a predicted probability per variable, $\hat {p}_{j}$. Note that inclusion of the intercept *α*_0_ in (2) guarantees that $\sum _{j=1}^{P} \hat {p}_{j} = 1$, as desired. The logistic regression establishes a marginal relationship between *v*_*jk*_ and *X*_*jc*_. For modeling *V*_*j*_, first note that *v*_*jk*_ contains two types of dependencies: (1) a dependency between splits *k* for a given variable *j*, e.g. only one variable can be chosen per split; (2) dependency between variables and therefore, between their selection frequencies (*V*_*j*_). The first dependency is addressed by using a quasi-binomial likelihood qBin(*V*_*j*_;***α***,*τ*) for $V_{j} = \sum _{k} v_{jk}$, which allows for an over- or under-dispersion parameter *τ* by modeling Var(*V*_*j*_)=*τ* *p*_*j*_(1−*p*_*j*_) [11]. We do not explicitly address the second type of dependency, which implies that the estimation is based on a pseudo-log-likelihood:

3 $$ \left(\hat{\boldsymbol{\alpha}},\hat{\tau}\right)=\max_{\boldsymbol{\alpha},\tau}\left[\sum_{j=1}^{P} \log\left(\text{qBin}\left(V_{j}; \boldsymbol{\alpha},\tau\right)\right.\right].  $$

As a result the uncertainties of the estimates and the *p*-values of the co-data model do not have a classical interpretation, and cannot directly be used for inference. We are, however, primarily interested in the point estimates, $\hat {p}_{j}$, obtained by substituting $\hat {\boldsymbol {\alpha }}$ into (2), which are used to re-weigh variables:

4 $$ w_{j} = \left(\hat{p}_{j} - \gamma p^{0}\right)^{+}.  $$

As earlier, *γ* can be set to 1 which provides a natural cut-off for *p* ^0^, or *γ* may be tuned to more or less sparsity. Finally, we normalize *w*_*j*_ to obtain the sampling probabilities $\tilde {w_{j}}=w_{j}/\sum _{j} w_{j}$, which are then used to re-train the RF.

The relationships we are interested in are often non-linear, e.g. for external *p*-values the difference between 10^−4^ and 10^−2^ may be more relevant than that between 0.25 and 0.50. We therefore extend the linear model (2) to include more flexible modeling of continuous co-data with a monotonous effect, which is often natural and desirable. For that, we fit a generalized additive model with a shape constrained P-spline (SCOP, [12]), as implemented in the R package scam. Then, Eq. (2) becomes

5 $$ \text{logit}(p_{j}) = \alpha_{0} + \sum_{c=1}^{C_{1}} X^{n}_{jc}\alpha_{c} + \sum_{d=1}^{C_{2}} f_{d}\left(X^{c}_{jd}\right)  $$

where *X* ^*n*^ (*X* ^*c*^) denotes the sub-matrix of *X* containing the nominal (continuous) co-data, and *f*_*d*_() represents a flexible function provided by the SCOP. To model *f*_*d*_, SCOP uses $m(x)=\sum _{\ell =1}^{q} \theta _{\ell }B_{\ell }(x)$, where *B*_*ℓ*_ is a B-spline basis function, which is monotonously increasing when *θ*_*ℓ*_≥*θ*_*ℓ*−1_,*ℓ*=1,…,*q* [12]. The monotony in ***θ*** is enforced by defining $\boldsymbol {\theta } =\Sigma \tilde {\boldsymbol {\theta }}$, where $\tilde {\boldsymbol {\theta }} = [\tilde {\theta }_{1},\exp (\tilde {\theta }_{2}),\ldots,,\exp (\tilde {\theta }_{q})]^{T}$ and *Σ*_*rs*_=0 if *r*<*s* and *Σ*_*rs*_=1 if $r \geqslant s$. Smoothness is enforced by penalisation of the squared differences analogous to [13]. Setting *Σ*_*rs*_=−1 for $r \geqslant s$ renders a monotonically decreasing spline. Unrestricted splines can in principle also be used in the co-data model, but are more liable to over-fitting.

Instead of using the default of *γ*=1, this parameter can be tuned by a grid search. This requires calculating *w*_*j*_ and refitting a RF for each grid-value of *γ*. The optimal value of *γ* is then the one with the best oob performance. Note that tuning *γ* with the oob predictions may result in a degree of optimism. This may be solved by embedding the procedure in a cross-validation loop. When *γ* is not tuned, the oob performance of CoRF may also be slightly optimistic, because the primary data was used to estimate the weights (4). However, when the regression model (2) is parsimonious, the overoptimism is likely small, as verified empirically in the Application section. To ensure that the co-data model is parsimonious, it may be useful to perform co-data selection to remove redundant co-data sources, which also assists in assessing the relevance of the co-data. The Additional file 1 supplies an heuristic procedure to do so.

### CoRF algorithm

The CoRF procedure may by summarized as follows:

1. Fit a base RF with uniform sampling probabilities and obtain *v*_*jk*_.
2. To disentangle the contributions of the various co-data sources.
  Fit co-data model (2), if only linear effects are assumed.
  Fit co-data model (5) with shape constrained P-spline(s), if flexible, monotone effects are required.
  Optionally: exclude redundant co-data sources and re-fit the co-data model.
3. Obtain the predicted probabilities $\hat {p}_{j}$ from the fitted co-data model.
4. Calculate the sampling probabilities *w*_*j*_ with threshold parameter *γ*. Default is to set *γ*=1, optionally *γ* can be tuned.
5. Refit the RF for each vector of $\tilde {w_{j}}$.
6.
  If *γ* is not tuned (i.e. *γ* = 1), we directly obtain the CoRF, the base RF and their oob performances.
  If *γ* is tuned, obtain $\hat {\gamma }$ by maximizing the oob performance. Tuning *γ* may introduce a bias in the oob performance. Hence, the entire procedure is cross-validated when *γ*-tuning is employed.

### Implementation

The method as described here is implemented in a corresponding R package, called CoRF, and is available on GitHub. It depends on the R package randomForestSRC for fitting the RF [14–16]. A feature of this package that is of key importance for CoRF is the option to assign a sampling probability per variable. In addition, randomForestSRC applies to regression, classification and survival analysis, and by extension, so does CoRF.

For classification by the RF the recommended minimal node size is one. The node size can be tuned [17], but a RF is not very sensitive to the minimal node size. In CoRF the quality of the selected variables may influence the fit of the co-data model. Variables that are used higher up in a tree are, on average, more relevant, and variables that split a node of size 2 are the least relevant. For CoRF, we believe it is better to slightly increase the minimal node size, improving the quality of the selected variables and as a result improve the quality of the co-data model. As default in CoRF, we set the minimal node size for classification at 2.

Generally, CoRF will need a larger number of trees to fit than a base RF. A base RF needs enough trees to capture the underlying signal in the data. CoRF additionally needs an indication of the relevance of each variable, which feeds back to the co-data model. Also, a co-data model that contains splines generally needs more trees than a co-data model with only linear effects. In the LNM example, described below, we set the number of trees at 15.000 to ensure convergence of both the RF/CoRF and the co-data model. A lower number of trees, e.g. 2.000, gives a similar result in terms of predictive performance, but the variability between fits increases. When using CoRF, we recommend to use at least 5.000 trees to ensure a reliable, good fit of the co-data model. An additional advantage of a large number of trees with tuning is that the variability between RF fits decreases, allowing for a more reliably selection of *γ*.

A RF is a computationally efficient algorithm to use for high dimensional data, primarily because at each node it selects only from $\sqrt P$ variables. CoRF inherits this efficiency and when the default *γ* = 1 is used, only one RF refit is needed. Next to (re)fitting the RF, the only additional computation required for CoRF consists of fitting the co-data model. Further tuning of *γ* may improve the performance, but also requires i) refitting a RF for each value for *γ*, and ii) an additional cross-validation loop to assess performance, thereby increasing computational cost considerably.

### Evaluating predictive performance

The predictive performance of CoRF and other classifiers was assessed on oob samples by two metrics: i) the area-under-the-roc curve (AUC; [18]); and ii) the Brier score [19]. AUC is based on ranks and evaluates *discrimination*. It combines sensitivity and specificity, which are both important in a clinical setting. Moreover, it is a good indicator for the performance of a RF with unbalanced data [18]. Brier score is based on residuals and evaluates *calibration*. It equals the average Brier residual, i.e. *B*_*i*_=(*Y*_*i*_−*q*_*i*_)^2^, where *q*_*i*_ is the fraction of trees predicting *Y*_*i*_=1. Brier score is reported in a relative sense, with the base RF as benchmark. For comparing CoRF with RF we implemented significance testing, both for *Δ*AUC: the difference between AUCs, using R’s pROC package [20] and for *Δ*Brier: the difference between Brier scores, using the one-sided Wilcoxon signed-rank test on paired Brier residuals $\left (B^{\text {RF}}_{i}, B^{\text {CoRF}}_{i}\right)$. When performance was evaluated on the same data as used for training, we used multiple 2/3 - 1/3 splits, meaning that the power for testing, for which only 1/3 of the samples can be used, can be limited. These splits result in multiple *p*-values, which we aggregate by applying the median, which was proven to control the type I error rate under mild conditions [21].

Similarly to the base RF, CoRF automatically renders oob predictions. CoRF is an empirical Bayes-type classifier, which uses the relation between the co-data and the primary data to estimate sampling weights. Such double use of data could lead so some degree over overoptimism, although this will likely be limited given that the co-data model is parsimonious. In addition, when splines were used, the effective degrees-of-freedom were reduced by imposing monotony. Nevertheless, in the examples below, we verified the oob performance of CoRF by cross-validation when training and evaluation was applied on the same data.

### Comparable methods

To our knowledge, there is only one high-dimensional prediction method that can explicitly take *multiple* sources of co-data into account: the group-regularized (logistic) ridge (GRridge [3]). CoRF provides several conceptual advantages over GRridge. First, unlike CoRF, GRridge requires discretisation of continuous co-data. Second, CoRF fits the co-data coefficients in one model, (2), instead of using the co-data sources iteratively. Third, CoRF is computationally more efficient, because it a) inherits the better computational scalability of RF with respect to *P*; and b) requires very little tuning and no iterations. Finally, as with a base RF, CoRF is naturally able to incorporate categorical outcomes with > 2 groups (as demonstrated in the cervical cancer example). GRridge inherits the advantages of ridge regression, e.g. better interpretability of the model and the ability to include mandatory covariates. In the Application section we compare the performances of these two methods for the LNM example.

## Applications

### Predicting Lymph node metastasis with TCGA data

To exemplify the CoRF method, we use it to predict the presence of a lymph node metastasis (LNM) for patients with HPV negative oral cancer using RNASeqv2 data from TCGA [22]. We focus on the HPV-negatives, because these constitute the majority (approx. 90%) of the oral cancers, and HPV-positive tumors are known to have a different genomic etiology [23]. Early detection of LNM is important for assigning the appropriate treatment. Diagnosis of LNM with genomic markers could potentially improve diagnosis and treatment [24].

The primary data consists of normalized TCGA RNASeqv2 profiles of head-and-neck squamous cell carcinomas (HNSCC), which were downloaded together with the matching normalized DNA copy number co-data from Broad GDAC Firehose using the R package TCGA2STAT. Of the 279 patients described in [22], we used the subset of 133 patients that had HPV-negative tumors in the oral cavity. Of these patients, 76 presented a LNM and 57 did not.

To enhance the prediction of the base RF, we consider three types of co-data in this example: (1) DNA copy number; (2) *p*-values from the external microarray data GSE30788/GSE85446; (3) a previously identified gene signature [24–26]. These three types of co-data demonstrate the variety of co-data sources that can be included in CoRF. The DNA copy number data are measurements on the same patients. We use the cis-correlations between DNA copy number and the RNASeqv2 data. Given the nature of RNASeqv2 and DNA copy number data (discrete and ordinal, respectively), we applied Kendall’s *τ* to calculate the correlations, giving *τ*_*j*_,*j*=1,…,*P*. Note that the DNA data are only used during training of the predictor; these are *not* required for test samples, which distinguishes this type of predictor from integrative predictors [27]. The *p*-values of GSE30788/GSE85446 are derived from measurements of the same type of genomics features (mRNA gene expression), but measured on a different platform (microarray) than that of the primary RNAseq data and on a different set of patients. The gene signature is a published set of genes that were found to be important in a different study. Figure 1 illustrates how the various types of data are used within CoRF.

Each type of co-data has its own characteristics that needs to be taken into account in the co-data model. For the DNA copy number data, we a priori expect that a gene with a positive cis-correlation is more likely to be of importance to the tumor [28]. We use a monotonically increasing spline *f*_1_ to model the relation between *p*_*j*_ and $X^{c}_{j1} = \tau _{j}$ (5). For the *p*-values of GSE30788/GSE85446, we a priori expect that genes with a low *p*-value are more likely to be important on the TCGA data, and we thus use a monotonically decreasing spline *f*_2_ to model the relation between *p*_*j*_ and $X^{c}_{j2} = \text {pval}_{j}$. The third type of co-data, consisting of the published gene signature is included in the co-data model (2) as a binary variable: $X^{n}_{j1}=1$ when gene *j* is part of the signature, and 0 otherwise.

Data set GSE30788/GSE85446 consists of 150 Dutch patients (of which 60 presented a LNM and 90 did not) with a HPV-negative oral cancer tumor who are in that respect similar to the TCGA patients. Gene expression was measured by microarray, the *p*-values on GSE30788/ GSE85446 were calculated using a Welch two-sample t-test; further details on the study can be found in [29]. The differences between the TCGA and the Dutch data (notably the platform and the geographical location of the patients) preclude a straightforward meta-analytic data integration. Also, our focus here is on the TCGA data, which were measured on a more modern platform, but shared genomic features with the Dutch co-data may enhance the weighted predictions.

After training the base RF and the CoRF, we validate these classifiers on an independent data set (GSE84846). GSE84846 contains microarray expression data of 97 HPV-negative oral cancer patients from Italy, of whom 49 had a LNM [29]. To directly apply the classifiers to the validation data, we need to account for the differences in scale between RNASeqv2 and microarray data. First, the TCGA RNASeqv2 data are transformed by the Anscombe transformation (i.e. $\sqrt (x+3/8)$). Next, both the TCGA RNASeqv2 and GSE84846 data are scaled to have zero mean and unit variance. We only included genes that could uniquely be matched between the two data sets (leaving 12838 genes). Since this validation does not require any re-training, the performance is directly assessed by comparing the predictions with the actual labels. As an alternative to this validation, we also use the relative frequency of variables used by the base RF and CoRF on the TCGA data as sampling probabilities in training a new RF on GSE84846 data, in which case the oob-performance was used.

We also assess the performance of the base RF and CoRF in terms of variable selection on both the training and validation data sets. For the TCGA training data set, we first select a set of genes (based on *V*_*j*_), retrain on this subset, and assess the performance with a 10-fold cross-validation. For the validation data set, we first select variables on the TCGA training data with the variable-hunt-vimp (vh-vimp) as described by [30]. Roughly, vimp measures the importance of a variable by assessing the decrease in predictive performance when the values of the variable are ‘noised up’, e.g. randomly permuted across samples. Then, we refit with the selected set of variables on the TCGA training data, and evaluate the performance of the refitted model on the validation data using oob-performance. To assess the stability of variable selection with a base RF and CoRF, we repeatedly (20 times) sample 84 out of 133 TCGA cases without replacement and fit a base RF and CoRF to each sampled set. Note that the sampling fraction mimics the expected fraction of independent samples in a re-sampling scheme, 0.632. We preferred subsampling over resampling here, because the latter would lead to duplicate samples in the sampled set. Stability of gene selection was then assessed by calculating the average overlap between any combination of two sets of variables selected from the subsampled training sets, varying the sizes of the selection sets from 10,20,…,100 genes.

### Performance on LNM example

**Fig. 2:**
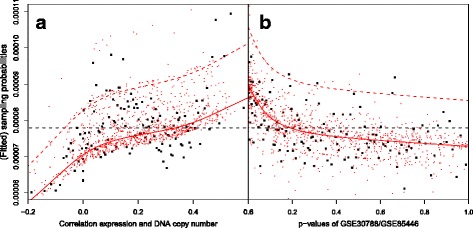
Fit of the co-data model for the LNM example. Each square represents 100 genes grouped by either (**a**) DNA copy number-expression correlation or (**b**) *p*-value. The red lines represent the marginal fit across the correlations or *p*-values. The top red lines represent the fit for genes present in the gene profile. The cloud of red dots represent the fitted values for 1000 randomly selected genes

**Fig. 3:**
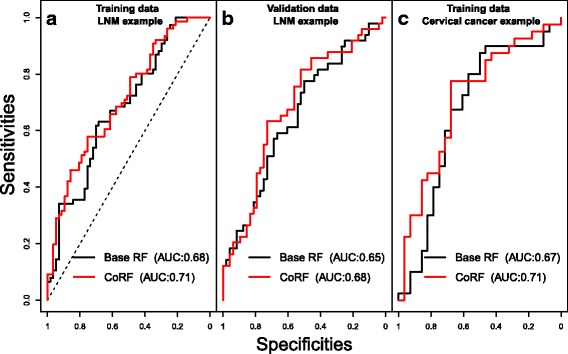
The ROC curve based on oob predictions for the base RF and CoRF. The ROC curve based on oob predictions for the base RF and CoRF; (**a**) the TCGA training data, (**b**) validation data set (GSE84846), and (**c**) The cervical cancer example

By examining the fit of the co-data model (Fig. 2), we observe that $\hat {p}_{j}$ is estimated higher for genes with a high cis-correlation, for genes with a low *p*-value on GSE30788/GSE85446, and for genes that are present in the gene signature. By prioritising these genes we observe an improvement in oob-AUC (base RF: 0.682, CoRF: 0.706) and a relative decrease in oob-Brier score of 2.7%. Figure 3 a and b show the ROC-curves (specificity versus sensitivity), parametrized by a threshold for the proportion of trees predicting LNM. When assessing significance using ten 2/3 - 1/3 splits, rendering an effective test sample size of *n*_test_=*n*/3≈44, the median p-values equal $\tilde {p}_{\Delta \text {AUC}} = 0.255$ and $\tilde {p}_{\Delta \text {Brier}} = 0.034$, hence significant at *α*=0.05 for the latter. In terms of Brier residuals, predictions improved for 27.2 out of 44 test samples, on average across splits. With 10-fold cross-validation we also see an improvement by using CoRF (cv-AUC base RF: 0.675, CoRF: 0.690). On the validation data we find that CoRF renders a slightly larger improvement (AUC base RF: 0.652, CoRF: 0.682); here the Brier score decreases by 2.6%. In this case we may use all 97 samples for significance testing. Then, the resulting *p*-values are: *p*_*Δ*AUC_=0.056 and *p*_*Δ*Brier_=0.0048, hence close to significant for the first and significant for the latter metric. In terms of Brier residuals, predictions improved for 64 out of 97 samples.

**Fig. 4:**
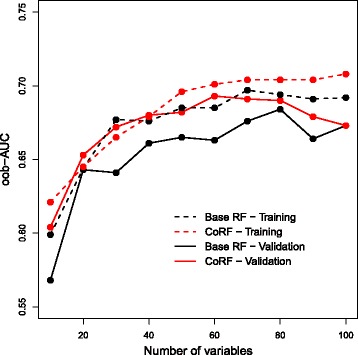
The performance of RF/CoRF for given numbers of variables selected with vh-vimp for the LNM example. For the (TCGA) training data the performance was assessed by a 10-fold cross-validation. For the validation data set (GSE84846) the prediction models where directly applied

Retraining on the validation data using only the sampling probabilities derived from either the base RF/CoRF fits to the TCGA data yields a similar result (oob-AUC base-RF: 0.656, CoRF: 0.695). From Fig. 4 we observe that CoRF also improves the oob-AUC when applying variable selection to both the training and validation data. Finally, stability of gene selection, when selecting genes with the vh-vimp measure, increased by 17%, averaged across sizes of the selection sets. This means that when selecting genes using random subsets of samples the overlap between two selected sets of equal size is on average substantially higher with CoRF than with RF. For gene selection with *V*_*j*_ the average stability increased by 36%. For these data, tuning of *γ* does not improve results, see Additional file 1.

For comparison with GRridge, we find a cv-AUC of 0.682 on the training data and AUC of 0.689 on the validation data. With GRridge the global penalty parameter of the ridge regression was estimated using a 10-fold cross-validation and performance on the training data was assessed using a second 10-fold cross-validation. For the validation data we directly applied the resulting classifier. In performance this is comparable to CoRF, but note that CoRF is quicker, especially when we want an estimate of the prediction error (see the “Computational time” section).

### Cervical cancer example

In this second example, our aim is to predict cervical (pre-)cancer on a very high-dimensional methylation data set. The methylation data consists of 365620 methylation sites, and contains 68 samples of which 28 correspond to normal cervical tissue, 36 have a high-grade precursor lesion (CIN3; CIN = cervical intraepithelial neoplasia) and 4 have cervical cancer. A diagnosis of either of the latter two stages usually implies surgery. The samples were taken using a self-sample test, implying a challenging diagnostic setting. The data used in this example originate from [31] where more information can be found on the clinical details and on the preprocessing of the methylation data. When training the RF, we consider the three separate categories, while for the final prediction we add up the votes for CIN3 and cancer, because of the small sample size for the latter. As before, our aim is to improve the prediction of the base RF by including co-data (e.g. CoRF).

**Fig. 5:**
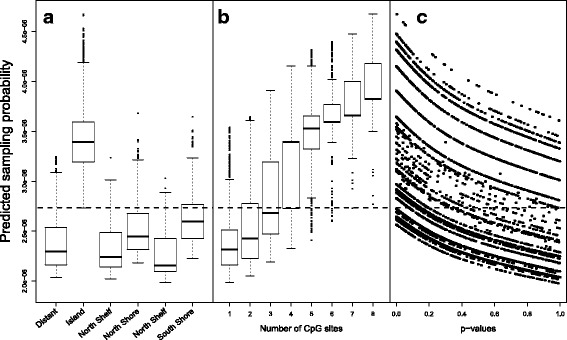
Fit of the co-data model for the cervical cancer example. Displayed are the estimated sampling probabilities for 10000 randomly selected methylation sites displayed by (**a**) location of the methylation site, (**b**) the number of CpGs, and (**c**) *p*-values. Figure c only displays the methylation sites that are up-regulated

The co-data consists of the location of the probe relative to the known CpG islands (categorized in 6 classes, including CpG-island), the number of CpG sites included in the genomic location targeted by the probe, and *p*-values, obtained by differential methylation analysis on a related, external study [32]. The latter study also compares methylation levels of normal cervical tissue (20) versus CIN3’s (17), but on surgically obtained cervical tissue. Hence, the setting is different than for our primary self-sample study, but these co-data are possibly very useful. The location of the probe is modeled as a nominal variable in the co-data model. From the fit of the co-data model (Fig. 5), we observe that the location of probe has as strong effect on $\hat {p}_{j}$, and indeed CpGs located within a CpG island are most important. The number of CpG sites can be either modeled as a factor variable or with a monotonically increasing spline. We opted for the second option, thus explicitly assuming that methylation sites with more CpG sites are likely more relevant, which seems reasonable when considering the fit of the co-data model (Fig. 5). Note that modeling the number of CpG sites as a factor variable gave a similar result.

Finally, for the external *p*-values we expect a different co-data effect for methylation sites that were either up- or down-regulated in the external co-data. We a priori expect a stronger effect of probes that are up-regulated, because down-regulated effects in the tissue data are hard to discover in self-samples, due to likely contamination of affected samples by adjacent normal tissue. We accommodate this distinction by modeling the interaction between the *p*-value and the direction of regulation (both obtained from the co-data), essentially fitting two monotonically decreasing splines, one for up- and one for down-regulated methylation sites. We indeed find that there is an effect of the *p*-values for the up-regulated methylation sites (Fig. 5). For the the *p*-values of the down-regulated methylation sites, the co-data model did indeed not identify an effect.

For this diagnostic setting, we observe there is an improvement by using CoRF (oob-AUC base RF: 0.666, CoRF: 0.710) and a decrease in oob-Brier score of 4.6%. Fig. 3 c shows the ROC-curves (specificity versus sensitivity), parametrized by a threshold for the proportion of trees predicting CIN3/Cancer. When using ten 2/3 - 1/3 splits, rendering an effective test sample size of only *n*_test_=*n*/3≈23, the median p-values equal $\tilde {p}_{\Delta \text {AUC}} = 0.351$ and $\tilde {p}_{\Delta \text {Brier}} = 0.065$, hence close to significant at *α*=0.05 for the latter. In terms of Brier residuals, predictions improved for 14.5 out of 23 test samples, on average across splits. With 10-fold cross-validation we find a similar increase in AUC (cv-AUC base RF: 0.661, CoRF: 0.702). In this case tuning rendered a value of *γ*=1.7, which excluded all but 105 variables. Tuning increased the cross-validated performance to AUC = 0.737 (See Additional file 1).

### Computational time

When using 5000 trees and without tuning of *γ*, the LNM example (*n*=133, *P*=12838) runs in 1:18 min (single threaded on a E5-2660 cpu with 128 gb memory). The cervical cancer example (*P*≈350.000,*n*=68) runs 22:24 min, of which the co-data model takes 8 min. By comparison, fitting a GRridge (R package GRridge) with a 10-fold cross-validation to estimate the global *λ* takes 2:07 min for the LNM example and 35:12 min for the cervical cancer example. To estimate the predictive error by cross-validation with GRridge the respective times need to be multiplied by the number of folds, rendering it much slower than the default CoRF (*γ*=1), which does not require cross-validation.

## Discussion and conclusion

The LNM and cervical cancer examples demonstrate that CoRF is able to improve the base RF by using co-data. Of course, the amount of improvement relates directly to the relevance of the co-data for the data at hand. The co-data are relevant if, for example, some of the co-data groups contain a relatively large number of variables related to the outcome, or if a continuous source of co-data (e.g. external *p*-values or correlations with other genomic measurement) correlates strongly with the importance of a variable. Figures 2 and 5 show the relevance of the co-data for our examples. Including additional informative co-data could further increase the performance of CoRF. Hence, expert knowledge on the domain and available external data is crucial. In addition, stability of the set of selected variables increased by the use of CoRF. We argue that the use of co-data provides a stronger foundation for the classifier, which may enhance generalization to other measurement platforms, which is sometimes problematic in omics settings.

If the co-data are non-relevant, CoRF provides a safeguard against over-fitting. Firstly, the co-data weights are estimated from a parsimonious (2) or smooth (5) model to ensure that they are stable. To further stimulate parsimony, one may conduct the co-data selection procedure described in the Additional file 1, which removes redundant sources of co-data. Practically, the use of CoRF is a bit more demanding than the use of RF: one needs to think about what co-data could be of use, and invest time in processing such co-data. On the other hand, this may also be perceived as an advantage: the classification process requires more involvement of the problem owner, e.g. a clinician or molecular biologist, instead of being a ‘black box’.

CoRF essentially aims at reducing the haystack of genomics variables by using co-data. Of course, one could also use ad-hoc filtering methods to preselect variables on the basis of existing information, but this introduces a level of subjectivity and sub-optimality when the threshold(s) are not chosen correctly. CoRF formalizes the weighting and thresholding process and lets the data decide on the importance of a given source of co-data. We expect CoRF to be most useful in (very) high-dimensional settings. In such settings, variables likely differ strongly in predictive ability while the size of the haystack complicates the search. In such situations our co-data approach can assist in identifying the relevant variables. For *P*<*n* settings, the prediction model is trained with a (relatively small) selected set of features. This means that i) learners not supported by co-data (e.g. base RF) are fairly well able to discriminate the important variables from the non-important ones; and ii) the small *P* complicates good estimation of our empirical Bayes-type (sampling) weights. Hence, in such a situation, CoRF (and co-data supported methods in general) are less likely to boost predictive performance. CoRF is weakly adaptive in that it learns the sampling weights from both the primary and the co-data, in contrast to other adaptive methods like the enriched RF [33] or the adaptive lasso [34], where weights are inferred only from the primary data. In high-dimensional applications such strong adaptation is more likely to lead to over-fitting, unlike the co-data moderated adaptation.

CoRF inherits its computational efficiency from the RF. When the tuning-free version is used (*γ*=1), we empirically found that the oob performance suffices and cross-validation is not required. This makes the methodology very suitable for applications with extremely large *P*. Tuning of *γ* may slightly improve the predictive performance, but at a substantial computational cost, given the required grid search for *γ* and the additional CV loop. The CoRF methodology may be combined with any bagging classifier that uses the random subspace method, such as a random glm [35] or a random lasso [36]. If variable selection is more stringent for a particular method (i.e. less noisy), then identification of the relationships of the co-data model may be easier. On the other hand, if most of the variables are not used, then we are unable to obtain an reliable assessment of the quality of those variables which may complicate fitting the co-data model. One possible extension of CoRF could be to use the depth at which variables are used by the RF, for example through the average or minimal depth [30]. Variables that are used higher up in a tree are, on average, more relevant, and it could be beneficial to assign larger weights to these variables in the co-data model. Another way of accomplishing this is to replace *v*_*ij*_ by a measure that counts how often each variable is used in classifying the oob samples, analogous to the intervention in prediction measure [37, 38]. This measure naturally up-weights variables that are often high up in a tree. We intend to investigate these matters in the future.

## Additional file

Additional file 1 Supplementary Files. Contains description about automatic co-data selection, results on tuning *γ* and evaluation of performance of CoRF with vimp. (PDF 288 kb)
