# Supplementary material for: Improved high-dimensional prediction with Random Forests by the use of co-data
Source: BMC Bioinformatics. 2017 Dec 28;18:584. doi: 10.1186/s12859-017-1993-1 (PMC5745983; doi:10.1186/s12859-017-1993-1)
Supplement: Additional file 1 — Supplementary Files. Contains description about automatic co-data selection, results on tuning γ and evaluation of performance of CoRF with vimp. (PDF 288 kb) [file 12859_2017_1993_MOESM1_ESM.pdf]

# Supplementary Information: Improved high-dimensional prediction with Random Forests by the use of co-data

Dennis E. te Beest[1], Steven W. Mes[2], Saskia M. Wilting[3], Ruud H. Brakenhoff[3], Mark A. van de Wiel[1,4]

1. Department of Epidemiology and Biostatistics, VU University Medical Center, Amsterdam, The Netherlands 2. Department of Otolaryngology-Head and Neck Surgery, VU University Medical Center, Amsterdam, The Netherlands 3. Department of Medical Oncology, Erasmus MC Cancer Institute, Erasmus University Medical Center, Rotterdam, The Netherlands 4. Department of Mathematics, VU University, Amsterdam, The Netherlands

# 1 Supplementary information

## 1.1 Co-data selection

As stated in the Main Document, the co-data model is based on a pseudo-likelihood and its $p$-values do not have the classical interpretation. Nevertheless, one can easily verify the distribution of this $p$-value under the hypothesis of random assignment of the co-data to the variables. In other words, similar to how variables can be permuted in gene set enrichment scores [2], one may permute the co-data labels to establish the distribution of the $p$-value under random assignment. Note that this is not an exact argument, because the null-hypothesis 'no association between $V_j$ and $X_{jc}$' does not guarantee exchangeability of the co-data labels. This $p$-value can easily be verified for any data example, because the permutations require only a re-fit of the co-data model, not a re-training of the RF. Logically, if there is no association between $V_j$ and $X_{jc}$, there is no effect of $X_{jc}$ on the CoRF fit. In practice, this means that one can safely remove a co-data source when its corresponding $p$-value is above a given threshold, e.g. 0.05, and conclude it is non-informative. Note that a low $p$-value, on other hand, does not guarantee a genuine relationship: the 'sample size' $P$ as used for the $p$-value calculation in the quasi-binomial model is not a genuine sample size (in terms of independent samples) given the potentially strong correlations between $V_j$.

In the LNM example we may, for example, want to give priority to genes that are present on the cancer census list (*http://cancer.sanger.ac.uk/census*, accessed April 2017). The cancer census list is a catalogue of genes that have been previously linked to cancer. In total 492 of these genes are present in the LNM example. We can incorporate this information with a binary variable indicating whether or not a gene is on this list. Through permutations we find

a $p$-value of 0.16, suggesting we can exclude the cancer census genes from the set of viable co-data. We also observed that the $p$-values resulting from the permutations are uniformly distributed and is identical to the asymptotic $p$-value from the co-data model suggesting that, at least in some situations, a permutation is not needed.

If CoRF would be fitted with a set of spurious co-data (despite its high p-value), $\hat{p}_j$ would be close to $1/P$ for all variables and CoRF essentially selects a random set of genes (for any $\gamma > 0$). This behavior is of course undesirable, hence the importance co-data screening, co-data model checking, and biological motivation. It is also possible that a set of co-data has a low p-value (and is not rejected), but does not improve oob-performance. In such a case it still is possible that variable selection is improved (verifiable with cross-validation) or there may be a compelling biological reasons to include the source of co-data. If neither is the case, it may be better to use the base RF (thus not using co-data). Note that in principle a proper $p$-value for the effect of the co-data can be calculated through permutation of outcome $Y_i$, but this requires refitting a RF and an co-data model for each permutation and is computationally heavy.

## 1.2 Tuning $\gamma$

The thresholding parameter $\gamma$ can be adjusted to accommodate for more or less sparse settings. For the LNM example we find that, after exploring a grid of $\gamma$ values, a $\gamma$ value of 1 maximizes the oob-AUC (Figure S1). For the methylation example we find that the oob-AUC is maximized for $\gamma$ value of 1.7, increasing the oob-AUC to 0.766 (Figure S1). In a 10-fold cross-validation we find an AUC of 0.737. This suggest that the oob-AUC is overoptimistic after tuning but, given the small sample size, a 10-fold cross-validation is likely to give an underestimated value.

Figure S1 suggest that oob-AUC is unimodel with respect to $\gamma$, although there are some stochastic fluctuations due the random nature of the RF. The reason for this unimodality is that, assuming there is not a complete correspondence between co-data predicted variable relevance and actual relevance, increasing $\gamma$ will at some point lead to "over-selecting" on the co-data. As a result, with an increasing $\gamma$, the oob-AUC at some point decreases. Note that $\gamma$ has an upper limit, when $\gamma >= max(\hat{p}_j)/p^0$ no variables are selected for refitting.

What can be considered as the best $\gamma$ depends on what the final aim of the RF is. If the fitted RF is directly going to be used for prediction, then the $\gamma$ that maximizes the oob-AUC is the best choice. If the aim is to do gene selection, a trade-off needs to be made between selection based on the co-data and selection based on the primary data. A high $\gamma$ puts more emphasis on selection with the co-data which, as a result, puts less emphasis on selection with the primary data (and vice versa for a low $\gamma$). For the LNM example, this is illustrated in Figure S2 where selection with a $\gamma$ value of 0.9 outperforms selection with a $\gamma$ value of 1.0. Selection with a $\gamma$ value of 1.1 on other hand performs poorly, suggesting too much emphasis was put on the co-data in the selection process.

## 1.3 CoRF with *vimp*

Instead of using $v_{jk}$, a different measure that seems intuitively appealing to use is the variable importance (*vimp*) [1]. Within the CoRF framework, we could replace $logit(p_j)$ by $vimp_j$, solve this model with linear regression to obtain $\hat{vimp}_j$. One issue here is that we lack a clear way to transform $\hat{vimp}$ to probabilities as these entities are intrinsically on a different scale (an ad-hoc solution would be to normalize $\hat{vimp}_j$ to obtain $\hat{p}_j$)). The main issue with *vimp* is that it is expensive to calculate, especially in high dimensional settings, and it is typically correlated with $V_j$ (Figure S3). As a result $\hat{p}_j$ and $\hat{vimp}$ are also strongly correlated (Figure S3) and thus will give a comparable performance, albeit at much greater computational costs.

# References


[1] L. Breiman. Random forests. *Machine Learning*, 45(1):5–32, 2001.

[2] A. Subramanian, P. Tamayo, V.K. Mootha, S. Mukherjee, B.L. Ebert, M.A. Gillette, A. Paulovich, S.L. Pomeroy, T.R. Golub, E.S. Lander, and J.P. Mesirov. Gene set enrichment analysis: A knowledge-based approach for interpreting genome-wide expression profiles. *Proceedings of the National Academy of Sciences*, 102(43):15545–15550, 2005.

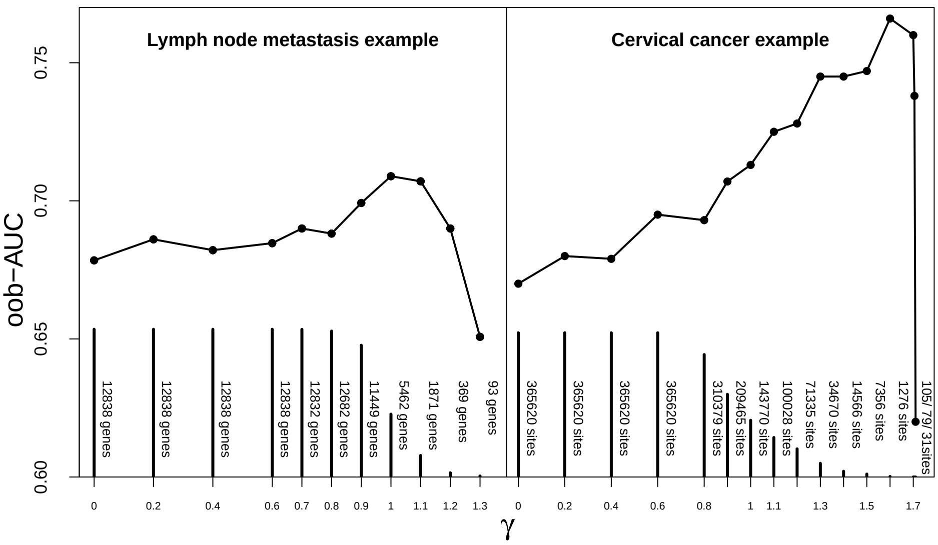


Figure 1: The oob-AUC for both data examples for a range of $\gamma$ values. The tuned $\gamma$ is the value that maximizes the oob-AUC. For the LNM example $\hat{\gamma} = 1$, and for the cervical cancer example $\hat{\gamma} = 1.7$. The bars indicate how many variables with non-zero sampling probability are left.

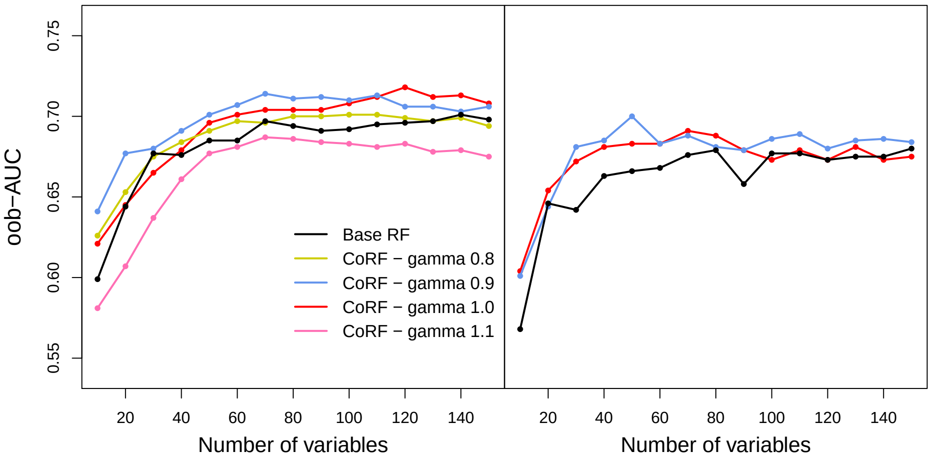


Figure 2: Performance of and base RF and CoRF for the LNM example on the the validation on data (GSE84846) as measured by the AUC for various selection sizes. In (A) genes were selected by the frequency with which they were used on the TCGA data. In (B) genes were selected based on vh-vimp measure.

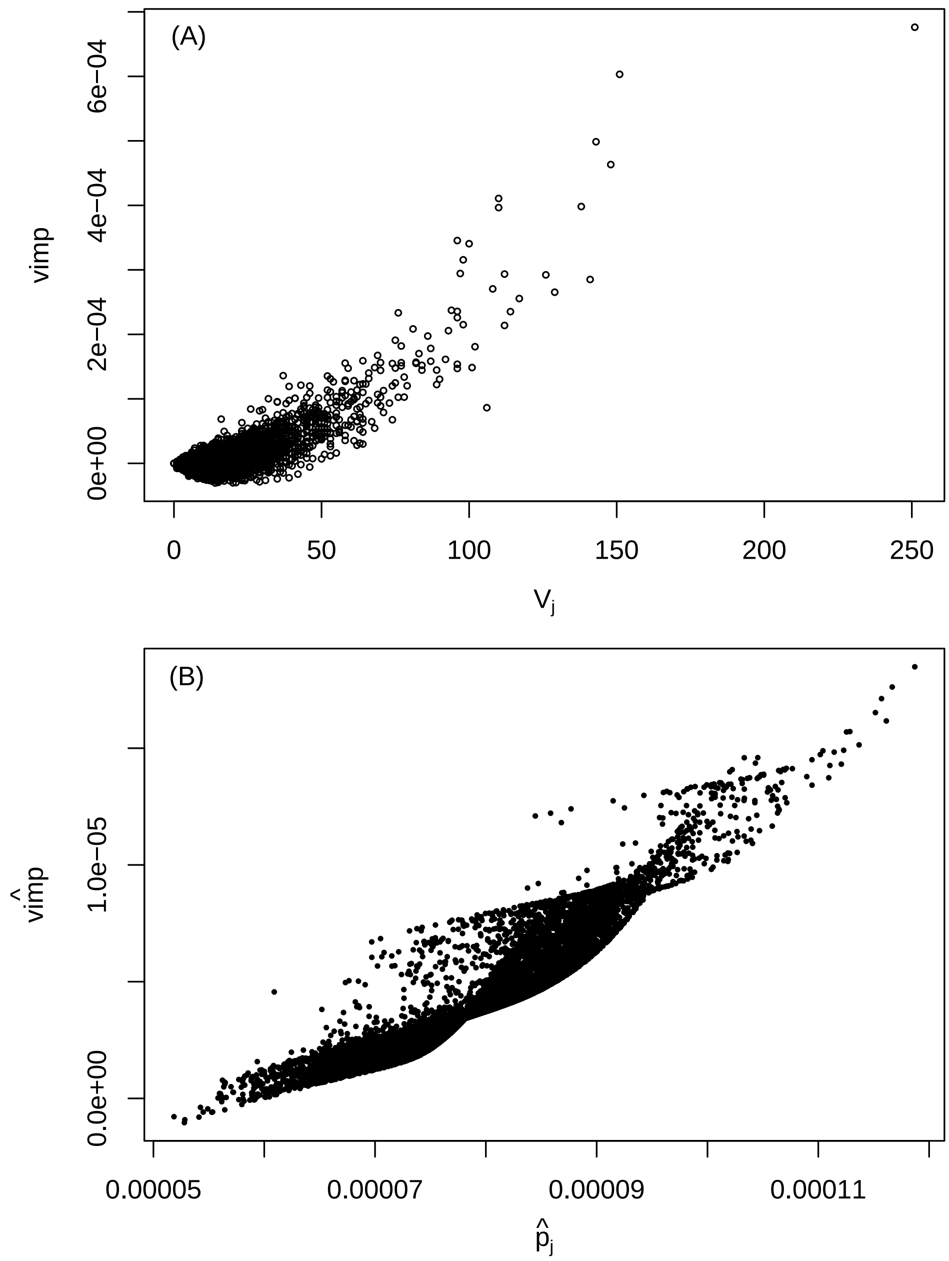


Figure 3: (A) The number of times each variables is used $V_j$ versus the *vimp* for the TCGA data of the LNM example, and (B) estimated with the co-data model, $\hat{p_{ij}}$ and $\hat{vimp}$.
